# COM4/392: In Vitro Diagnostic Net: Virtual community and marketplace of laboratory medicine

**DOI:** 10.2196/jmir.1.suppl1.e17

**Published:** 1999-09-19

**Authors:** M Dressel

**Affiliations:** ^1^IVD-net GmbH, FreitalGermany

**Keywords:** In-vitro Diagnostic, Laboratory Techniques and Procedures

## Abstract

**Introduction:**

Using the Internet for laboratory needs to quickly retrieve information, such as special parameters, addresses, market news & innovations or a product's manual, is time-consuming. There exist already numerous Web sites and their number is ever-increasing. But does the useful information presented in these sites always reach the desired target group? Evidence shows that this has not happened, so far. IVD-net has identified this problem and devoted a good deal of effort to resolving it, over the last year. As an alternative to time-consuming searches in a large number of sources, IVD-net offers a centralized, firmly established Internet forum devoted to in vitro diagnostics.

**Methods:**

IVD-net intends to be a guiding compass in the "jungle" of Web-pages dealing with laboratory medicine. This guidance is being created in a virtual community of specialists and interested people this field. It is the community of people connected by a common interest in in-vitro diagnostics. In this community, step-by-step, a network of knowledge and experiences is created, where each member can benefit from. IVD-net is authentic; there is no contribution without reference to the original source.

**Results:**

Structure of IVD-Web-sites:

- German and English languages.
- "public" pages for welcome and orientation.
- interactive "members'" pages for registered members.

Membership is free of charge. Members pages consist of:

- News: daily new information and scientific events.
- News Group: the bulletin board for your questions, answers, comments, information.
- Addresses : list of addresses and/or home pages of important partners in laboratory medicine directly linked to their URL.
- Archives: thematically arranged & handy for research.
- Hardware and Software: marketplace of instrumentation and a software catalogue. This is a virtual catalogue with efficient search functions, direct ordering options and attractive special offers of alternative suppliers!

**Discussion:**

Economic globalization and increasing pressure to reduce costs in public health systems bring up a great challenge; the one of finding new solutions.

